# Bridging the Gap Between Regulatory Approval and Patient Access: The Case for Insurance Coverage of Approved Allogeneic Stem Cell Therapy in Knee Osteoarthritis

**DOI:** 10.7759/cureus.114764

**Published:** 2026-08-18

**Authors:** Amit Kumar Agarwal, Gautam Das, Pradeep Aggarwal, Rajashekar Danda

**Affiliations:** 1 Department of Orthopaedics, Indraprastha Apollo Hospital, New Delhi, New Delhi, IND; 2 Department of Interventional Pain Management, Daradia Pain Hospital, Kolkata, IND; 3 Department of Orthopaedics and Joint Replacement Surgery, Shalby Multi-Specialty Hospitals, Mohali, IND; 4 Department of Orthopaedics, PRK Super Specialty Hospitals, Hyderabad, IND

**Keywords:** allogeneic mesenchymal stromal cells, health insurance coverage, knee osteoarthritis, patient access, regulatory approval

## Abstract

Knee osteoarthritis (OA) is a major cause of pain, disability, and impaired quality of life, with most current therapies offering only symptomatic relief and limited disease modification. Patients with Kellgren-Lawrence grade II-III disease often fall into a therapeutic gap between conservative care and surgery. The approval of Stempeucel (Stempeutics Research Pvt. Ltd., Bangalore, India), an allogeneic mesenchymal stromal cell therapy, by the Drugs Controller General of India/Central Drugs Standard Control Organization reflects available and growing clinical evidence for regenerative approaches, including sustained improvements in pain, stiffness, and physical function, along with signals of structural benefit such as increases in cartilage volume and thickness. However, insurance coverage in India remains inconsistent, labeling such therapy as “experimental” despite regulatory approval following clinical trial evidence. This disconnect highlights a critical gap between evidence-based authorization and reimbursement policies. Although uncertainties around long-term outcomes and cost-effectiveness persist, restricting access may limit public health benefits from obtaining such outcomes. An alignment of reimbursement frameworks with regulatory approval and clinical evidence, alongside continued data generation, is essential to ensure equitable access and facilitate responsible integration of regenerative therapies into OA management.

## Editorial

Introduction

Knee osteoarthritis (OA) is a major contributor to chronic pain, long-term disability, and impaired quality of life globally [1]. Despite a wide range of pharmacological and non-pharmacological interventions provide meaningful clinical benefit, options with potential structural or disease-modifying effects remain limited [2,3]. This limitation is particularly evident among patients with Kellgren-Lawrence (KL) grade II-III OA, who often continue to experience substantial symptoms despite conservative management, yet may not be ideal candidates for surgical intervention [3].

Regenerative medicine has emerged as a potential paradigm shift in the management of musculoskeletal disorders. Among these, allogeneic mesenchymal stromal cell (MSC) therapy has demonstrated anti-inflammatory, immunomodulatory and chondroprotective effects, maintaining cartilage volume and thickness [4,5]. In India, the approval of an allogeneic bone marrow-derived MSC therapy (Stempeucel; Stempeutics Research Pvt. Ltd., Bangalore, India) by the Drugs Controller General of India (DCGI)/ Central Drugs Standard Control Organization (CDSCO) following structured clinical development represents an important regulatory milestone [6]. Clinical and preclinical data, including randomized controlled trials (RCTs), have shown sustained improvements in patient-reported outcomes along with signals of structural benefit [4,5]. Furthermore, the recent European Society of Sports Traumatology, Knee Surgery and Arthroscopy - Orthobiologics Initiative (ESSKA-ORBIT) consensus has recognized cell-based therapies as a promising treatment option for KO while emphasizing the need for continued generation of real-world evidence to further define their optimal clinical use and long-term value [7].

However, in clinical practice this regulatory approval has not fully translated into real-world access, which is a critical gap of concern. Insurance providers in India have frequently categorized stem cell-based therapies as “experimental” or “investigational, even for regulator-approved stem cell therapies for their indications. This shows a potential area of divergence between regulatory authorization, clinical evidence and payer coverage policies. In this Editorial, we explore whether this disconnect reflects appropriate caution or a lag in policy adaptation, and examine its implications for patient access, health equity, and the adoption of regulator-approved disease-modifying therapies in OA.

An evidence-based, regulator-approved therapeutic option

Stempeucel, an allogeneic adult human bone marrow-derived, isolated, cultured, pooled and ex vivo expanded mesenchymal stromal/stem cell therapy (allogeneic BMMSCs), has undergone a comprehensive clinical development process prior to its approval by the DGCI under the CDSCO in February 2022 for the treatment of radiographic KL grade II-III knee OA [6]. Preclinical studies have demonstrated chondrogenic differentiation potential and the ability to promote cartilage repair, along with improvements in pain-related outcomes in OA models [8,9].

An exploratory Phase II randomized, placebo-controlled dose-finding trial demonstrated the safety and tolerability of intra-articular Stempeucel administration and identified the 25-million-cell dose as the most promising dose, showing consistent improvements in patient-reported outcomes. These findings informed the selection of the 25-million-cell dose for subsequent Phase III evaluation [8,9]. A multicentric, randomized, double-blind, placebo-controlled Phase III trial demonstrated significant and sustained improvements in Western Ontario and McMaster Universities Osteoarthritis Index (WOMAC) and Visual Analog Scale (VAS) outcomes versus placebo, with a between-group WOMAC total score difference of 632.74 points (95% CI: 473.91-791.57) at 12 months and 952.43 points (95% CI: 772.04-1132.82) at 24 months [4,5]. MRI T2 mapping demonstrated no worsening of cartilage degeneration in the Stempeucel arm over 24 months compared with gradual worsening in the placebo arm, suggesting chondroprotective effects and signals of structural benefit [4,5].

These findings have subsequently been considered within broader evidence reviews of orthobiologic therapies and allogeneic MSC interventions for KO, where allogeneic MSCs have been recognized as a promising therapeutic approach supported by RCT evidence, while acknowledging the need for additional long-term and comparative effectiveness data [10-12]. Collectively, these findings suggest potential benefits beyond symptomatic management, although continued long-term evaluation remains important.

Innovation in manufacturing: pooled allogeneic MSC technology

An important aspect of Stempeucel is its underlying manufacturing platform. It is derived from the bone marrows of three healthy adult donors, isolated, pooled and ex vivo expanded under Good Manufacturing Practice (GMP)-compliant conditions. It involves a standardized cell-banking and expansion process involving characterized master cell banks and working cell banks, in a WHO-GMP certified laboratory [13]. The patented pooling technology addresses a key limitation of single-donor cell products, namely biological variability. By pooling isolated MSCs of three donors, greater consistency in cellular characteristics and functional properties is achieved. Supporting research suggests that pooled MSC populations can demonstrate more stable biological performance and improved scalability, essential for broader clinical use and improved outcomes [14,15].

To our knowledge, Stempeucel represents the only approved allogeneic, bone marrow-derived, pooled MSC therapy for KO, supported by a development program that included preclinical evaluation, an exploratory randomized Phase II dose-finding study, and a multicenter, randomized, double-blind, placebo-controlled Phase III trial with 12-month and 24-month follow-up in patients with KL grade II-III knee OA [4-8]. This represents a notable step forward in the standardization of regenerative therapies and highlights the evolving Indian regulatory ecosystem to evaluate complex biologics. Despite this advancement, the gap in alignment with reimbursement policies creates a paradox. A scientifically validated and regulator-approved therapy, based on an advanced and scalable manufacturing platform, may remain inaccessible to many patients due to inconsistent reimbursement policies and denial of coverage by few payers, often related to classification of cell therapies as investigational or experimental.

Reconsidering the “experimental” classification: a policy perspective

A commonly cited reason for insurance denial is the classification of stem cell therapies as “experimental” or “investigational.” While this designation may historically have reflected limited evidence or unregulated practices, its continued application to regulator-approved therapies warrants closer examination. Regulatory approval by the DCGI is based on established evidence of safety, efficacy, and quality derived from structured clinical trials. In this context, it may be questioned whether therapies that have undergone Phase III clinical trial evaluation and received marketing authorization should be grouped with unapproved interventions.

Counterpoint: understanding payer caution

Despite regulatory approval and encouraging clinical evidence, some degree of payer caution is understandable. Questions regarding long-term effectiveness, real-world durability, and cost-effectiveness continue to evolve. In our opinion, the broad and heterogeneous use of stem cell-based interventions, particularly unapproved autologous cell therapies across multiple indications, may contribute to uncertainty in policy decisions by blurring distinctions between unapproved interventions and regulator-approved products. However, this should be considered a possible explanation rather than an established determinant of payer decision-making.

In this context, cautious reimbursement approaches may reflect a need for clearer differentiation between regulator-approved cell therapies and unapproved stem cell interventions. Establishing such frameworks could facilitate the generation of high-quality real-world evidence and enable more informed, confident, and sustainable coverage decisions.

Health economic and cost-effectiveness considerations

To our knowledge, robust published studies evaluating the cost-effectiveness, budget impact, quality-adjusted life-year (QALY) gains, and potential arthroplasty-aversion benefits of Stempeucel within the Indian healthcare setting are currently limited. Nevertheless, health-economic analyses from other healthcare systems suggest that such evaluations are feasible and informative. For example, a South Korean cost-effectiveness study evaluating allogeneic umbilical cord blood-derived MSC (hUCB-MSC) therapy for patients with KO-associated cartilage defects, compared with microfracture surgery, reported favorable incremental cost-effectiveness ratios and a high probability of meeting accepted willingness-to-pay thresholds [16]. While these findings cannot be directly extrapolated to Stempeucel or the Indian reimbursement environment because of differences in the cell product, treatment indication, comparator, healthcare system, and cost structure, they highlight the potential value of formal economic evaluation in informing coverage decisions for cell-based therapies.

The generation of India-specific health-economic evidence therefore remains an important next step in informing reimbursement policy and understanding the broader value of this therapy within routine clinical practice. At the same time, therapies that have undergone structured clinical development and received regulatory approval may merit ongoing reassessment within payer frameworks based on the evolving body of clinical, health-economic, and real-world evidence, rather than being categorized solely on the basis of historical perceptions associated with stem cell interventions. Expanding appropriate patient access through evidence-based reimbursement pathways could facilitate wider clinical adoption in carefully selected patients and support the generation of meaningful real-world data on long-term effectiveness, healthcare utilization, arthroplasty avoidance, patient-reported outcomes, and cost-effectiveness. Such data would further strengthen future reimbursement evaluations and help ensure that coverage decisions remain aligned with emerging clinical and economic evidence.

Global perspective and reimbursement considerations

Globally, several MSC-based therapies have received regulatory approval, including TEMCELL HS in Japan (JCR Pharmaceuticals Co., Ltd, Ashiya, Japan) and remestemcel-L (Ryoncil; Mesoblast, Inc., New York, NY, USA) and omidubicel (Omisirge; Gamida Cell Ltd., Boston, MA, USA) in the United States [17-20]. In these settings, such therapies have been incorporated into reimbursement frameworks, reflecting increasing alignment between regulatory pathways and access mechanisms (Table 1).

**Table 1:** Other approved stem cell therapies around the world, their indications, country and inclusion for insurance reimbursement USFDA: United States Food Drug and Drug Administration; MHLW: Japanese Ministry of Health Labour and Welfare

Stem cell therapy	Approved Indication	Country, Regulatory Authority	Approval Year	Inclusion into National Health Insurance Reimbursement List
TEMCELL® HS Inj. [18]	Acute graft-versus-host disease (aGVHD) following hematopoietic stem cell transplant	Japan, Ministry of Health, Labour and Welfare (MHLW)	2015	Listed [19]
Ryoncil® (remestemcel-L) [20]	Steroid-refractory acute GVHD in pediatric patients ≥2 months	United States, FDA	2024	Listed [21]
Omisirge® (omidubicel-onlv) [22,23]	Hematologic malignancies undergoing umbilical cord blood transplantation after myeloablative conditioning to reduce time to neutrophil recovery and infections	United States, FDA	2023	Listed [24,25]

While these therapies differ in their approved indications and operate within distinct healthcare systems, these examples illustrate how reimbursement frameworks can evolve alongside regulatory approvals, supported by evidence-generation and defined coverage criteria [18-25]. In contrast, the limited reimbursement of stem cell therapies in India may reflect the evolving nature of payer frameworks and reimbursement mechanisms for advanced therapies, as observed in clinical practice.

Implications for patient access and health equity

Insurance coverage is a key determinant of access to innovative therapies. When approved treatments are not reimbursed, access may depend on out-of-pocket payment, potentially widening healthcare burden and inequities. In KO, limited reimbursement of approved regenerative therapies may restrict access to non-surgical treatment options. Broader access could improve treatment availability and facilitate evaluation of their impact on disease progression, arthroplasty rates, and cost-effectiveness in real-world practice.

Conclusion

The availability of a regulator-approved allogeneic MSC therapy represents an important development in the management of KO. However, the gap between regulatory approval and insurance coverage highlights broader challenges in translating scientific innovation into patient access. While payer caution regarding long-term effectiveness and health-economic value is appropriate, approved therapies supported by evolving clinical evidence warrant transparent reassessment through established reimbursement frameworks rather than continued classification based solely on historical perceptions of stem cell interventions. Bridging this gap will require collaboration among regulators, clinicians, and payers, supported by ongoing evidence generation, including real-world outcomes and cost-effectiveness data. Such an approach can help ensure equitable access while maintaining rigorous standards of safety, effectiveness, and value.
